# CHN1 promotes epithelial–mesenchymal transition via the Akt/GSK-3β/Snail pathway in cervical carcinoma

**DOI:** 10.1186/s12967-021-02963-7

**Published:** 2021-07-08

**Authors:** Haoqi Zhao, Lan Wang, Shufang Wang, Xihua Chen, Min Liang, Xin Zhang, Jiedong Wang, Xiangbo Xu

**Affiliations:** 1 Graduate School of Peking Union Medical College, Chinese Academy of Medical Sciences & Peking Union Medical College, Beijing, 100730 China; 2 Reproductive Physiology Laboratory, National Research Institute for Family Planning, Beijing, 100081 China; 3 National Engineering and Research Center of Continuous Casting Technology, China Iron and Steel Research Institute Group, Beijing, 100081 China; 4 Biopharmaceutical R&D Center, Chinese Academy of Medical Sciences & Peking Union Medical College, Suzhou, 215126 Jiangsu China; 5 Department of Forensic Medicine, Xinxiang Medical University, Xinxiang, 453003 Henan China

**Keywords:** Cervical carcinoma, CHN1, Epithelial-mesenchymal transition (EMT), Akt/GSK-3β/Snail signaling pathway

## Abstract

**Background:**

Metastasis and invasion are crucial in determining the mortality of cervical carcinoma (CC) patients. The epithelial–mesenchymal transition (EMT) is now a universal explanation for the mechanisms of tumor metastasis. Α-chimeric protein (α-chimaerin, CHN1) plays an important role in the regulation of signal transduction and development. However, the molecular regulatory relationships between CHN1 and CC progression in relation to EMT have not yet been identified.

**Methods:**

The expression of CHN1 in CC tissues, adjacent tissues, and lymph node metastases from CC patients was detected by immunohistochemistry. Upregulation and knockdown of CHN1 were achieved by transfection of CC cells. The effect of CHN1 on cell proliferation was determined by CCK-8 and plate clone formation assays. Changes in migration and invasion capabilities were evaluated using scratch migration and transwell invasion assays. The effect of CHN1 overexpression and interference on xenograft tumor growth was determined by tumor weight and pathological analyses. The expression of EMT-related mRNAs was measured by qRT-PCR in transfected CC cells. EMT-related proteins and Akt/GSK-3β/Snail signaling pathway-related proteins were also evaluated by western blotting.

**Results:**

CHN1 was overexpressed in CC tissues and was associated with lymph node metastasis and low survival in CC patients. Overexpression of CHN1 promoted cell proliferation, migration, and invasion in CC cells. In contrast, silencing of CHN1 inhibited these phenomena. Overexpression of CHN1 promoted tumor formation in an in vivo xenograft tumor mouse model, with increased tumor volumes and weights. In addition, CHN1 induced the expression of EMT-related transcription factors, accompanied by the decreased expression of epithelial markers and increased expression of mesenchymal markers. The Akt/GSK-3β/Snail signaling pathway was activated by overexpression of CHN1 in vitro, and activation of this pathway was inhibited by the signaling pathway inhibitor LY294002.

**Conclusion:**

These results suggest that CHN1 promotes the development and progression of cervical carcinoma via the Akt/GSK-3β/Snail pathway by inducing EMT.

**Supplementary Information:**

The online version contains supplementary material available at 10.1186/s12967-021-02963-7.

## Background

Cervical carcinoma is one of the main malignancies that threatens the health of women worldwide [1]. With the development of early diagnostics and treatments for cervical carcinoma, the incidence of cervical carcinoma in developed countries has declined significantly [2], but the incidence and mortality of cervical carcinoma among young women in developing countries remain high [1, 3].

The metastatic spread of cancer cells from the primary site to distant areas, such as the lymph nodes around the vessels of the pelvic wall, is the major reason for the unsuccessful treatment of cervical cancer [4, 5]. Accumulated evidence demonstrates that the epithelial-mesenchymal transition (EMT) is responsible for the invasion and metastasis of various carcinomas and has been associated with elevated resistance to chemotherapy and immunotherapy [6–8]. Despite significant efforts for verifying whether EMT reprogramming of tumor epithelial cells depends on the systematic occurrence at multiple regulation levels [6, 9], the exact underlying mechanisms connecting EMT, metastasis, and cervical carcinoma remain to be elucidated for a better understanding of cancer progression and the related therapeutic methods.

Chimaerin (CHN1) is a Ras-related Rho GTPase-activating protein (RhoGAP) involved in cytoskeletal regulation [10–12]. Two chimaerin isoforms, α and β, exist in mammalian genomes, each of which consists of at least two splice variants: a full-length type 1 transcript and a truncated type 2 transcript. Although both transcripts have a cysteine-rich domain followed by a GAP domain, type 2 isoforms are characterized by an SH2 domain-encompassing N-terminal [13–15]. CHN1 is widely investigated in neurobiology and is pivotal in neuronal signal transduction, brain development, synaptogenesis, and cognitive ability [12, 16–19]. It is also involved in the regulation of T cell adhesion and chemotaxis [20], transmission of signals in tumor progression by connecting cell adhesion and MAP kinase activation in the *Drosophila melanogaster* model [21], maintenance of epithelial morphology through its function as an apical-specific Rac1 GTPase activating protein [22], and key regulation of oculomotor axon guidance decisions in zebrafish [23]. CHN1 is well investigated in Duane retraction syndrome (DRS), in which *CHN1* mutations have been identified in patients [19, 24–26], and it is believed to have a crucial developmental function in this disorder. However, limited information on CHN1 has been reported in relation to cancer. Couch et al. showed that CHN1 could be used as a biomarker for the diagnosis of esophageal squamous dysplasia and squamous cell cancer [27]; Sun et al., however, claimed that CHN1 could be used as a novel prognostic marker for diffuse large B-cell lymphoma [28]. Previously, Liu et al. showed that CHN1 was highly expressed in human cervical cancer tissues and was positively regulated by *miR-205*; furthermore, high expression of CHN1 was also correlated with lymph node metastasis [29].

The aim of this study was to further investigate the role of CHN1 in the progression and metastasis of cervical carcinoma, and to reveal the potential mechanism by which CHN1 exerts its function. In the present study, we demonstrated that CHN1 is highly expressed in cervical carcinoma and is correlated with long-term prognosis. CHN1 can accelerate the occurrence of tumor biological behaviors and may stimulate the activation of EMT through the Akt/GSK-3β/Snail pathway to promote the metastasis of cervical carcinoma.

## Methods

### Tissue samples and patients

Two paraffin-embedded tissue microarrays (TMA), one consisting of 62 paired CC/adjacent non-carcinoma samples and another containing 84 CCs and eight lymph nodes from metastatic cervical carcinoma samples, were obtained from Shanghai Outdo Biotech (Shanghai, China) and Alena Bio (Xi’an, China), respectively. The other paraffin-embedded CC samples were histopathologically diagnosed and obtained from the PLA 251 Hospital. These patients did not undergo radiation and chemotherapy before surgery, and the samples were stored at − 80 °C.

### Cell lines, cell culture, and gene transfection

The CC cell lines, SiHa and HeLa, used in this study were purchased from the Cell Resource Center of the Peking Union Medical College (Beijing, China). The cells were cultured in DMEM/F12 medium (Thermo Fisher Scientific, USA) supplemented with 10% fetal bovine serum (FBS; Thermo Fisher Scientific) and maintained at 37 °C in a humidified atmosphere containing 5% CO_2_. The recombinant full-length *CHN1* gene expression plasmid and the *CHN1* shRNA-interference plasmid were synthesized and obtained from GenePharma (Shanghai, China). Cells were transfected with empty vector plasmids as negative controls. Transfection was performed in a 24-well plate (Corning Life Science, USA) using Lipofectamine 3000 (Invitrogen, USA) following the manufacturer’s instructions, and the efficacy of transfection was assessed by western blotting. Stable transfection clones were selected using 400 µg/mL G418 (Sigma, USA) for 14 days.

### Immunohistochemistry (IHC) and hematoxylin & eosin (H&E) staining

The paraffin-embedded sections were de-waxed, hydrated, and antigen retrieved in 0.01 M citrate solution at 95 °C for 20 min. They were then treated with 3% hydrogen peroxide for 10 min to block endogenous peroxidase activity. Mouse anti-human CHN1 monoclonal antibody (1:150, Abcam, UK) and rabbit anti-human Snail polyclonal antibody (1:500, Proteintech Group, USA) were used for incubation, followed by serum blocking. A PV-6002 kit (ZSGB-Bio, Beijing, China) was used in the following immunodetection. The specimens were observed and captured using an inverted microscope (Leica, Wetzlar, Germany). Tumors harvested from the xenograft nude mice were fixed and embedded in paraffin, and their pathological features were analyzed by H&E staining (Solarbio Life Science, Beijing, China).

### RNA extraction, reverse transcription, and quantitative real-time PCR (qRT-PCR)

Total RNA was isolated from cultured cells using TRIzol reagent (Invitrogen) and prepared according to the manufacturer’s instructions. The complementary DNA was reverse transcribed using the PrimeScript RT reagent kit (TaKaRa, China), and qPCR was performed with the SYBR Premix Ex Taq II (Tli RNaseH Plus) Kit (TaKaRa) using an ABI 7500 Real-time PCR system (Applied Biosystems, USA). All genes were analyzed in triplicate, and relative expression was normalized to that of the housekeeping gene β-actin. Relative quantity of each gene was calculated as 2^−ΔΔCt^. The primers used are listed in Additional file 1: Table S1.

### Western blotting

Equivalent extracted amounts of protein were separated with polyacrylamide SDS gels (SDS-PAGE) and transferred to polyvinylidene fluoride (PVDF) membranes (Thermo Fisher Scientific). They were then probed with different primary antibodies, including rabbit anti-CHN1 (1:150, Abcam), mouse anti-fibronectin (1:350, Invitrogen), rabbit anti-β-catenin (1:1000, CST, USA), rabbit anti-Snail (1:500, CST), rabbit anti-vimentin (1:1000, Proteintech Group), rabbit anti-E-cadherin (1:5000, Proteintech Group), rabbit anti-p-Akt (1:2000, CST), rabbit anti-Akt (1:1000, CST), and rabbit anti-p-GSK-3β (1:1000, CST), and the blots were detected with peroxidase-conjugated secondary antibodies (ZSGB-Bio). Signals were acquired using a chemiluminescence kit (Merck Millipore, Germany). Rabbit anti-β-tubulin (1:10,000) and mouse-anti GAPDH (1:10,000, both from Transgen Biotech, China) were used as internal controls. Universal anti-mouse/rabbit secondary antibodies were purchased from ZSGB-Bio (1:4000). To inhibit the PI3K/Akt pathway, the cells were treated with 5 μM PI3K inhibitor LY294002 (MedChem Express, USA) for 12 h.

### Immunofluorescence (IF) analysis

The cells were seeded on poly-l-lysine-coated (ZSGB-Bio) coverslips and fixed after culturing. After fixation with 4% paraformaldehyde solution at room temperature, the cells were washed with 0.01 M PBS solution three times. The cells were incubated with 5% goat serum blocking solution containing 0.3% Triton X-100 for 30 min. IF was performed in a wet box by overnight incubation at 4 °C with primary antibodies against CHN1 (1:150, Abcam), E-cadherin (1:200, Proteintech Group), and vimentin (1:300, Proteintech Group). Alexa Fluor® 597-conjugated secondary antibody (1:300, ZSGB-Bio) was used for incubation at 37 °C for 1 h after washing the cells with 0.01 M PBS. The cell nuclei were stained with DAPI (0.2 µg/mL, Sigma), and images were acquired using an inverted fluorescence microscope (Leica).

### Cell proliferation and migration assays

Cellular growth curves and colony-forming assays were used to evaluate cell proliferation rates. Briefly, cells were seeded, cultured, and incubated with the CCK-8 kit (Dojindo, Japan) reagent, and the optical density (OD) value was analyzed using a Synergy HT multi-mode reader (BioTek, USA) at 450 nm. A plate colony-forming assay was performed by seeding 200 cells/well in a culture dish. The cells were fixed, stained with crystal violet (Beyotime, China), and counted under a microscope (> 50 clones validated; Leica) after 14 days of culture.

Cell migration was evaluated by wound healing and transwell assays. Twenty-four hours after cell seeding and culturing, a scratch was made using a 200 μL sterile pipette tip. The cells were cultured in FBS-free medium, and wound confluence was observed after 24 and 48 h. The transwell assay was also used to confirm cell migration. A 2 × 10^4^ single-cell suspension was added into the upper chambers of 24-well transwell plates (Corning Life Science), and 0.1% FBS-containing medium was added to the lower chambers. The migrated cells were fixed, stained with crystal violet (Beyotime), and counted under a microscope after 24 h incubation.

### In vivo detection

For xenograft detection, 4-week-old NU/NU female nude mice were used (purchased from Vital River Laboratory Animal Technology Co., Ltd, China). SiHa cells (5 × 10^7^) were mixed with Matrigel (Becton Dickinson) and subcutaneously injected into the oxter of each mouse. Five mice were used in each group. Mice were anesthetized and sacrificed after 8 weeks, and tumors were harvested, measured, and stained. All the mice used in this study were bred at the SPF Animal Laboratory of the Institute of Science and Technology, National Research Institute for Family Planning.

### Statistical analysis

Statistics software (SPSS 18.0, IBM Corporation, USA) was used for data analysis. Data are expressed as the mean ± standard deviation (SD) after at least three independent experiments. Pearson’s chi-square test was used for clinical correlation analysis, and the Kaplan–Meier method with log-rank test was used to analyze the overall survival rate of cervical cancer patients. The differences between groups for the other experiments were assessed using one-way analysis of variance (ANOVA) and the Student–Newman–Keuls method. Statistical significance was set at *p* < 0.05.

## Results

### Overexpression of CHN1 in CC was related to metastasis and was negatively correlated with the overall survival time of patients

**Fig. 1:**
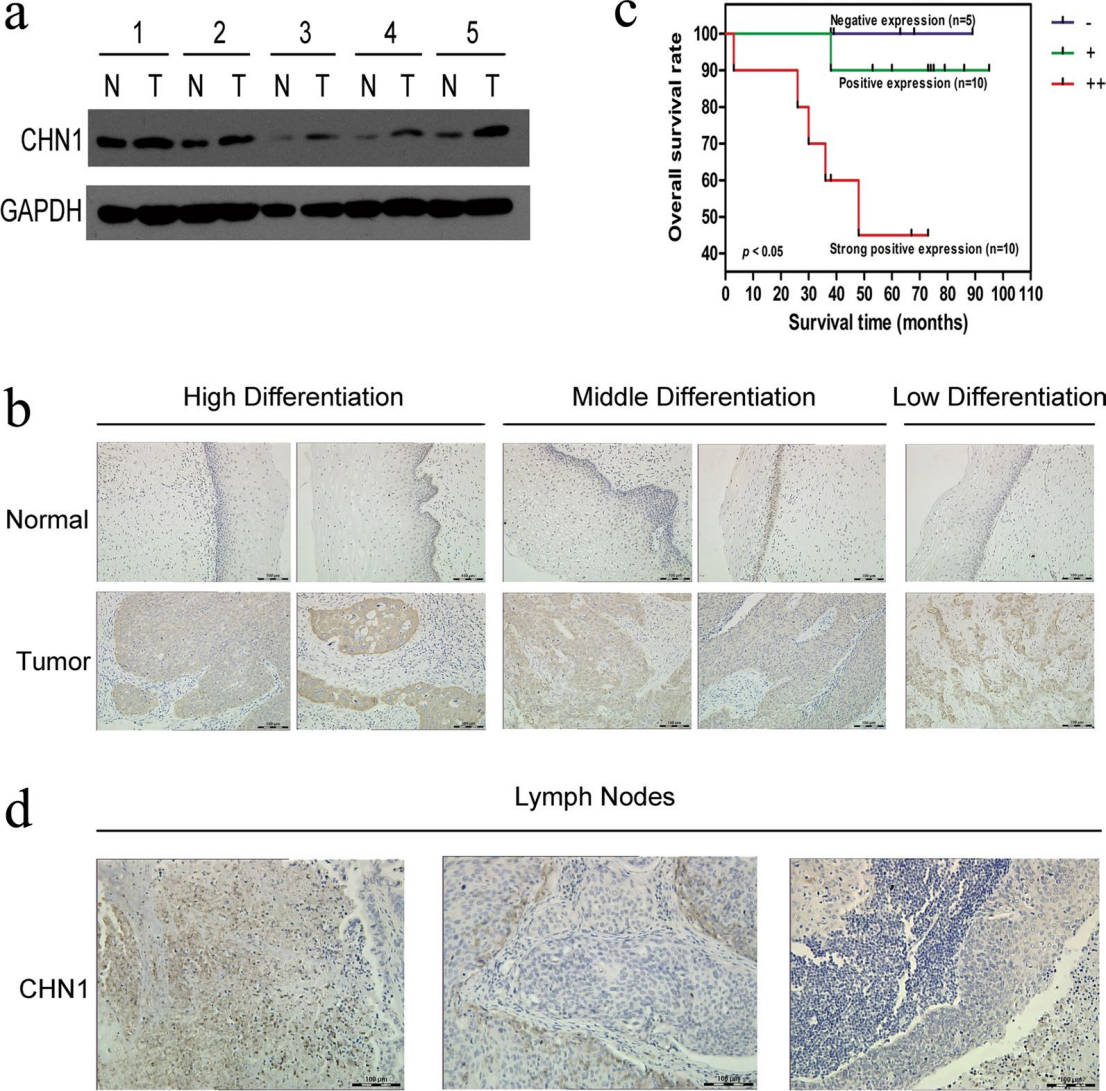
CHN1 is overexpressed in CC tissues. **a** Expression levels of CHN1 in CC and matched adjacent normal tissues. Three independent experiments were performed and GAPDH was used as the internal control. **b** IHC staining assays were performed to analyze the expression of CHN1 in CC with various differentiation states and matched non-carcinoma tissues (original magnification, ×100). **c** The Kaplan–Meier method with the log-rank test (*p* value) was used to estimate overall survival rates for CC patients with differential expression of CHN1. The blue line (−) indicates the survival rate of patients with negative expression of CHN1 (n = 5), the green line (+) indicates the survival rate of patients with positive expression of CHN1 (n = 10), and the red line (++) indicates the survival rate of patients with strongly positive expression of CHN1 (n = 10). **d** CHN1 was highly expressed in the metastatic lymph nodes of cervical carcinoma tissues (original magnification, ×100)

Western blotting was used to test CHN1 expression level in both CC and paracancerous tissues. In five pairs of CC and matched adjacent tissues, CHN1 was found highly expressed in tumors compared with that in the corresponding non-carcinoma tissues (Fig. 1a). IF staining (Additional file 2: Fig. S1a) and IHC assays (Additional file 2: Fig. S1b) showed that CHN1 was mainly concentrated in the cytoplasm. IHC was further performed to confirm the expression status of CHN1 in 62 paired CC/adjacent non-carcinoma TMA, and three representative pairs are shown (Fig. S1c). Among these samples, strongly positive expression of CHN1 was clearly observed in high-, medium-, and low-differentiation CC tissues (Fig. 1b), with an overall positive rate of 85.5% (53/62), which was notably different from the rate in non-carcinoma tissues (43.5%, 27/62; Table 1). However, CHN1 was mainly expressed in the basal cells of adjacent non-carcinoma epithelium tissues, due to rapid cell division and active cell proliferation (Fig. 1b). To further study the patient survival time and CHN1 status, we collected another 65 samples from CC patients for IHC detection; 25 patients participated in the follow-up study for the assessment of survival time. The overall survival rate varied among patients with differentially expressed CHN1. Compared to the group with negative expression of CHN1, strongly positive and positive expression of CHN1 were related to short overall survival times, with the shortest survival times in the strongly positive group (Fig. 1c). The median survival time of the strongly positive patients was 48 months. These data demonstrated that overexpression of CHN1 might negatively affect the overall survival time of patients.

**Table 1:** CHN1 expression levels in carcinoma and non-carcinoma tissues

Tissues	N	CHN1 expression			Positive rate (%)
		−	+	++	
Non-carcinoma	62	35	26	1	43.5
Carcinoma	62	9	31	22	85.5**

^**^P < 0.01 VS non-carcinoma tissues

Pearson *chi-*square test was performed to determine the statistical significance of the level of expression of CHN1 in cervical carcinoma and non-carcinoma tissues. – represents a negative expression of CHN1, + represents a strong expression of CHN1, and ++ represents a strong expression of CHN1

Subsequently, the clinicopathological significance of CHN1 expression was analyzed by chi-squared test in the 128 CC tissue assay. CHN1 level was related to lymph node metastasis (*p* = 0.003), but not with the age, clinical stage, or histological differentiation of patients (Table 2). Since pelvic lymph node metastasis is one of the common types of cervical cancer progression, we then detected the correlation between the metastasis of lymph nodes and CHN1 expression by IHC. CHN1 was found to be highly expressed in the metastatic lymph nodes of cervical cancer tissues, which indicated that CHN1 might be related to cervical cancer metastasis (Fig. 1d).

**Table 2:** Relationship between CHN1 expression and clinicopathologic feature

Features	Classification	N	CHN1expression		*P* value
			Low (%)	High (%)	
Age (years)	< 50	83	15 (18.1%)	68 (87.9%)	0.79
	≥ 50	45	9 (20.0%)	36 (80.0%)	
TNM stage	I	31	8 (25.8%)	23 (74.2%)	0.138
	II	42	10 (23.8%)	32 (76.2%)	
	III–IV	55	6 (10.9%)	49 (89.1%)	
Differentiation grade	Well	16	1 (6.3%)	15 (93.7%)	0.131
	Moderate	97	22 (22.7%)	75 (77.3%)	
	Poor	15	1 (66.7%)	14 (93.3%)	
Lymph node metastasis	No	78	21 (26.9%)	57 (65.4%)	0.003**
	Yes	50	3 (6.0%)	47 (94.0%)	

^*^^*^Statistical significance (*p* < 0.01) was shown in bold

### Up-regulation of CHN1 promotes the proliferation of CC cells in vitro and enhances tumorigenicity in vivo

**Fig. 2:**
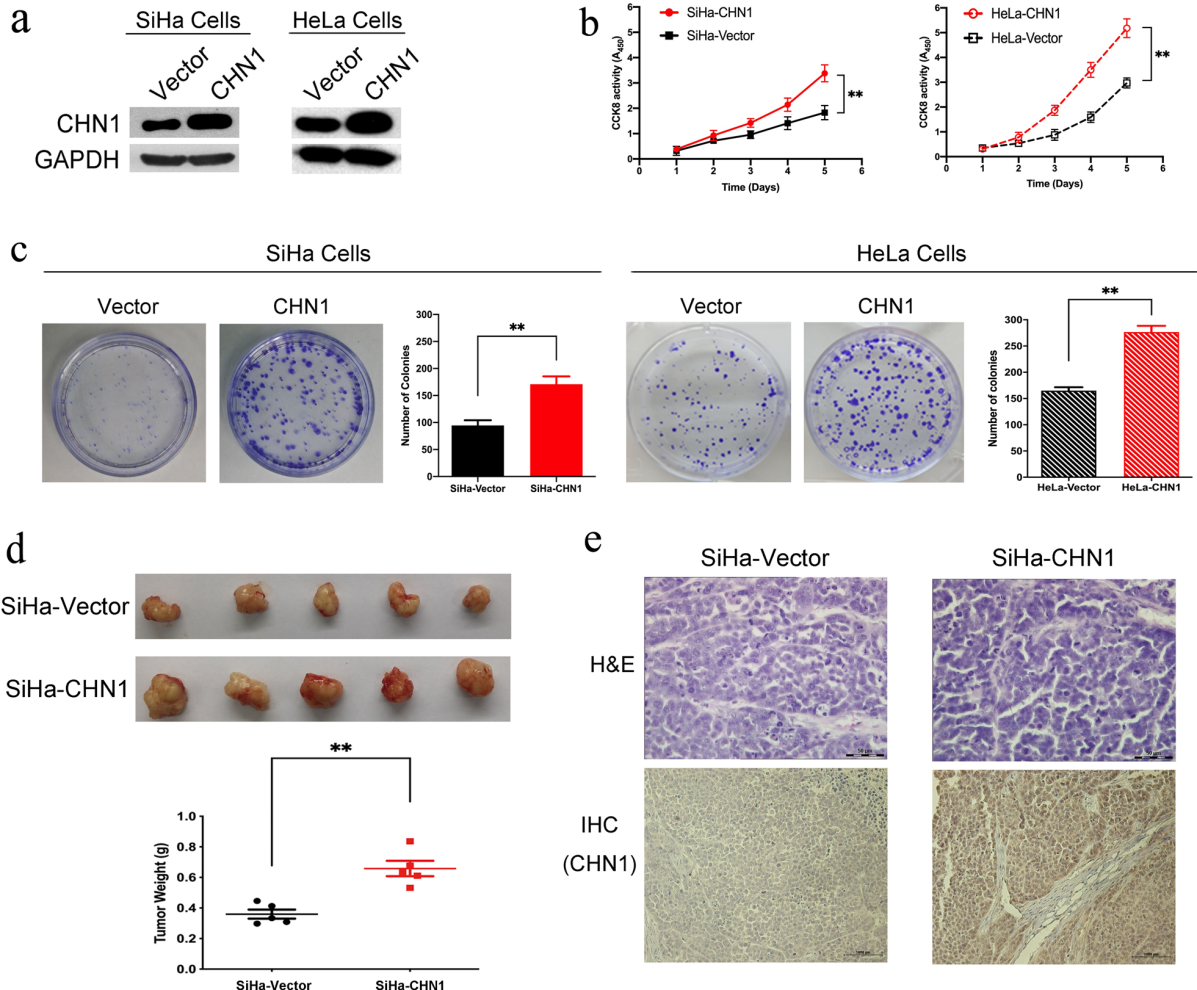
CHN1 overexpression promotes tumorigenicity in CC cells. **a** CHN1 was overexpressed in SiHa and Hela cells after transfection. GAPDH was used as the internal control. **b** Cell proliferation was enhanced after the overexpression of CHN1 in SiHa and Hela cells (data represent means ± SD, n = 3, **p < 0.01). **c** Foci formation was effectively promoted after CHN1 overexpression in SiHa and Hela cells (data represent means ± SD, n = 3, **p < 0.01). **d** Xenograft tumors were formed following SiHa-CHN1 and SiHa-Vector transfection in nude mice. 5 × 10^7 CHN1 overexpressed SiHa cells (SiHa-CHN1) or empty vector transfected SiHa cells (SiHa-Vector) were used for xenograft, respectively. Tumors were harvested and assessed 8-week post xenograft (right, n = 5 for each group, **p < 0.01). **e** Representative H&E and IHC staining for CHN1 expression in CHN1- and empty vector-transfected xenograft tumors (original magnification, ×200)

As CHN1 was highly expressed in cervical carcinoma tissues, the influence of its upregulation on cell proliferation was then tested by establishing a CHN1-high expression model in the SiHa and HeLa cell lines. As confirmed by western blotting, CHN1-overexpressed cells were successfully obtained, with vector-only transfected cells as the control (Fig. 2a). These cells were tested for their ability to proliferate both in vitro and in vivo. Using the CCK-8 assay, an increase in CHN1 level coupled with significantly enhanced cell proliferation was observed in the CHN1-overexpressed cells, but not in the vector-transfected group (Fig. 2b). Similarly, the colony-forming assay further verified that the number of colonies in CHN1-overexpressed cells was much higher than that in the vector group (Fig. 2c). These data demonstrated that overexpression of CHN1 may increase the proliferative capacity of cells in vitro. In addition, the tumorigenicity of SiHa-CHN1 and SiHa-vector was evaluated in nude mice, to test their proliferative ability in vivo. Eight weeks post-xenograft, tumors were observed in both groups. However, the tumors formed by the CHN1-upregulated SiHa cells showed significantly higher weights than those in the vector group (*p* = 0.001, Fig. 2d). H&E staining confirmed that the tumors consisting of these two kinds of cells had absent cell polarity in the parenchyma, enlarged cell nuclei, pathological mitoses, and apparent cell heteromorphisms, all distinctive features of tumor cells (Fig. 2e, upper panel). In contrast to the SiHa-vector group, additional IHC data showed that CHN1 was positively expressed in SiHa-CHN1-formed tumors (Fig. 2e, bottom panel), which implied that the formation of xenograft tumors might be caused by upregulation of CHN1. Taken together, these data demonstrated that increased CHN1 protein levels may promote cell proliferation both in vitro and in vivo.

### Down-regulation of *CHN1* inhibits the proliferation of CC cells in vitro and reduces tumorigenicity in vivo

**Fig. 3:**
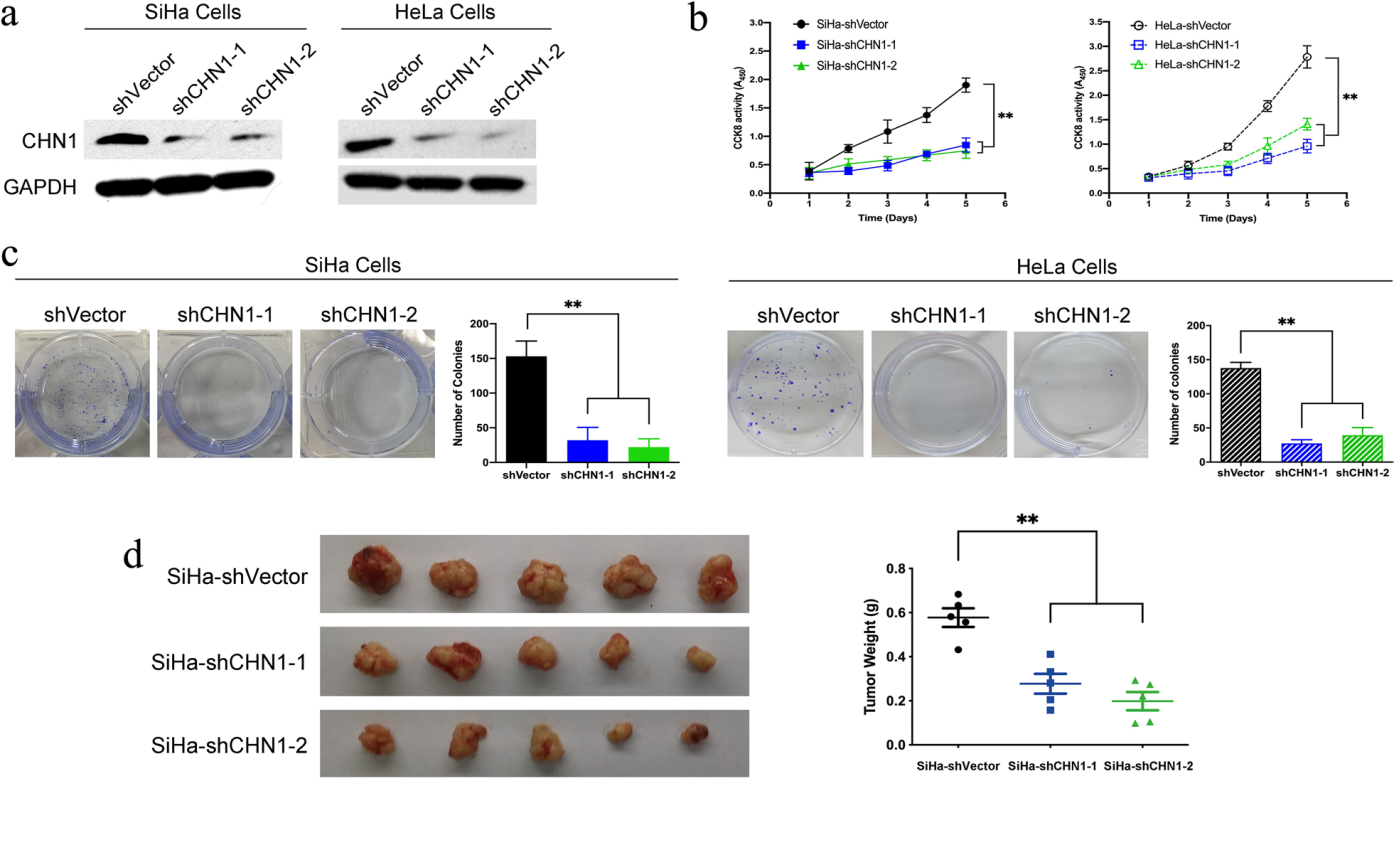
CHN1 interference inhibits the tumorigenicity of CC cells. **a** CHN1 expression was reduced after shRNA knockdown in both SiHa and HeLa cells. **b** Cell proliferation was inhibited after CHN1 knockdown in both cell lines (data represent means ± SD, n = 3, **p < 0.01). **c** Foci formation was inhibited after the interference of CHN1 expression in SiHa and Hela cells (data represent means ± SD, n = 3, **p < 0.01). **d** Smaller xenograft tumors were generated after the injection of SiHa-shCHN1 cells into nude mice. 5 × 10^7 CHN1-inhibited or empty vector transduced SiHa cells were used for in vivo detection, respectively. Tumors were harvested and assessed 8-week post xenograft (right, n = 5 for each group, **p < 0.01)

In addition to the upregulation experiments, *CHN1* interference was performed using two *CHN1*-targeted shRNAs, shCHN1-1 and shCHN1-2, with scrambled shRNA as a negative control (shVector), and cell proliferation was again assessed both in vitro and in vivo. Western blotting showed that the protein level of CHN1 was strongly decreased in both shCHN1-1 and shCHN1-2 groups, compared to the shVector group (Fig. 3a). Meanwhile, the CCK-8 assay showed that the proliferation of CHN1-downregulated cells (Fig. 3b), as well as their colony-forming ability (Fig. 3c), were significantly inhibited. The SiHa-shCHN1 and SiHa-Vector cells were used for in vivo tumorigenicity assay. This detection further confirmed that even though tumors developed in all three groups, the ones observed in the shCHN1-1 and shCHN1-2 groups were significantly smaller than those in shVector group (Fig. 3d).

### Interference of *CHN1* inhibits the invasion and migration of CC cells

**Fig. 4:**
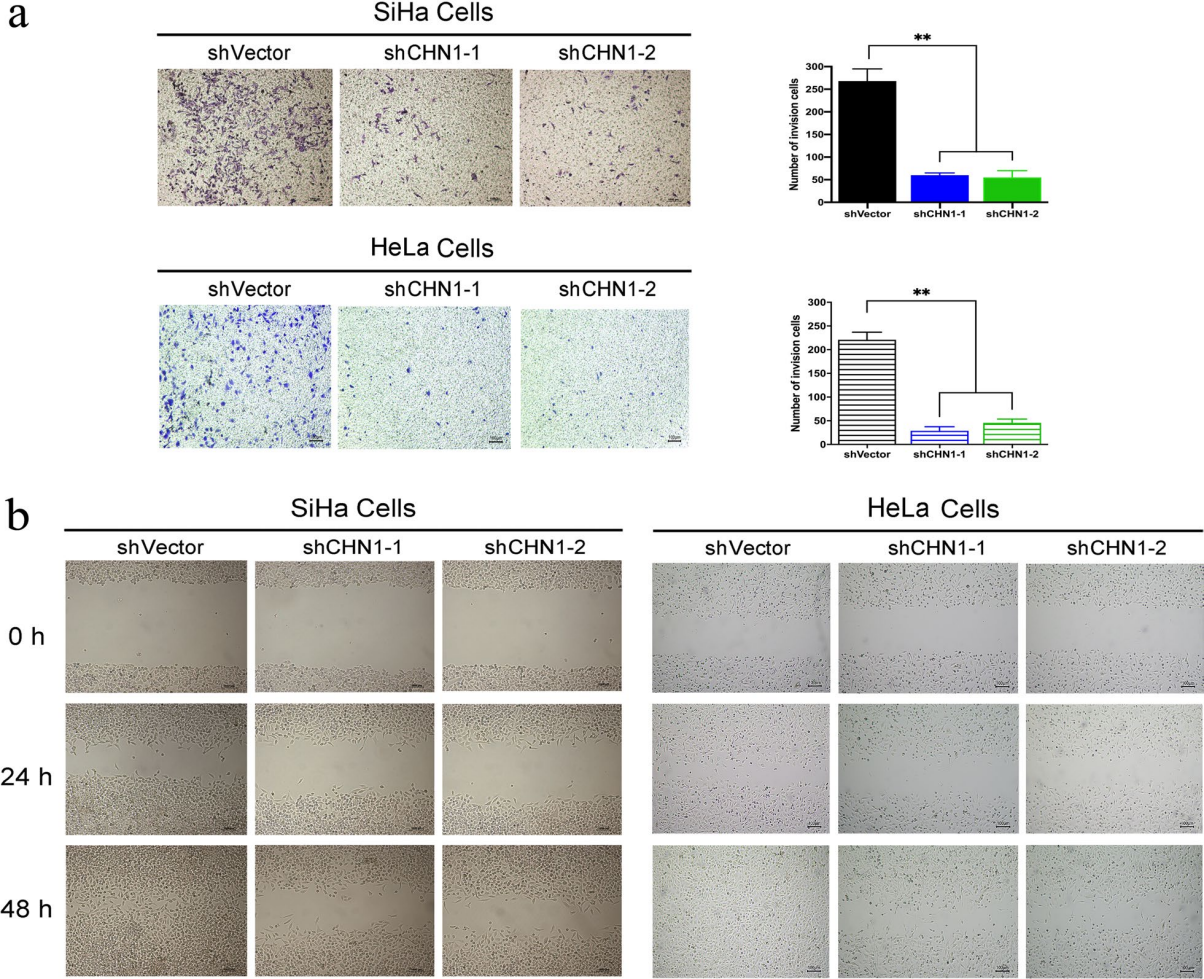
CHN1 interference decreases the invasion and migration of CC cells. **a** Transwell invasion assay detected inhibited invasion of SiHa and Hela cells after CHN1 interference. **b** Wound-healing assays detected reduced migration rates after down-regulation of CHN1 (original magnification, ×40). All data represent means ± SD, n = 3, **p < 0.01

Cell migration and invasion were assessed simultaneously in *CHN1*-downregulated CC cells. The transwell assay showed that cell invasion was significantly inhibited when *CHN1* was downregulated (Fig. 4a), and the wound-healing assay showed that cell migration was affected by the decreased expression of *CHN1* (Fig. 4b).

Taken together, these results demonstrated that the invasion and migration of CC cells can be inhibited by the downregulation of CHN1 in vitro.

### *CHN1* induces EMT though the Akt/GSK-3β/Snail pathway

**Fig. 5:**
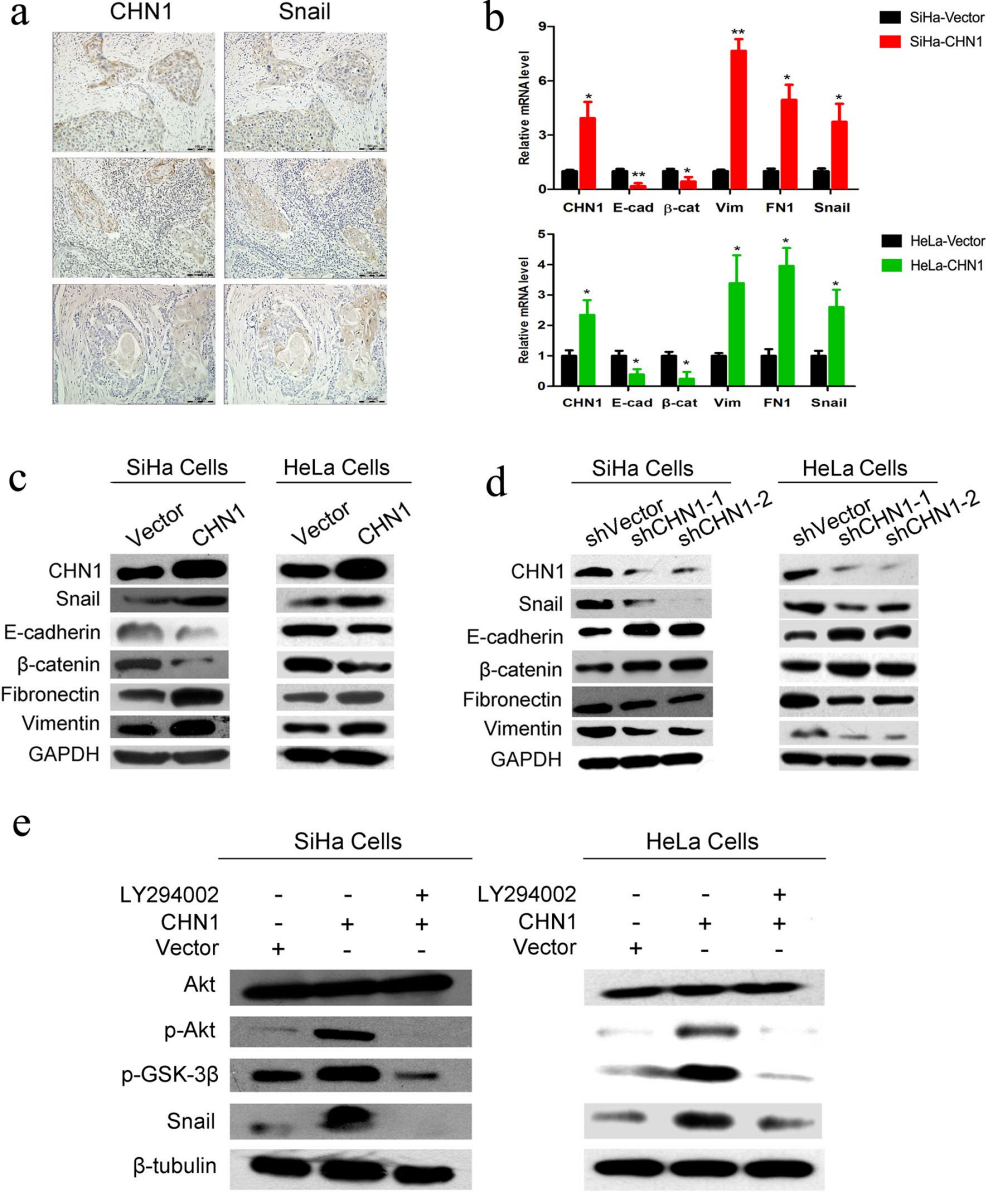
CHN1 expression induces EMT through activation of the Akt/GSK-3β/Snail pathway. **a** A representative expression of CHN1 and Snail in serial sections of cervical tumor tissues from three CC patients. An overlapped expression pattern of these two proteins is presented (original magnification, ×100). **b** Gene expression of CHN1, E-cadherin, β-catenin, vimentin, fibronectin, and Snail, in CHN1-upregulated SiHa and Hela cells and control vector-transfected cells, was assessed using qRT-PCR. The expression of EMT markers and EMT-related transcription factors was affected after up-regulation of CHN1 in SiHa and Hela cells. Data represent means ± SD, n = 3; *p* < 0.05. Overexpression (**c**) and interference (**d**) of CHN1 affected the expression of epithelial and mesenchymal markers. **e** Overexpression of CHN1 increased the expression of p-Akt, p-GSK-3β, and Snail. Treatment with Akt inhibitor LY294002 (5 μM for 12 h) reversed the impact of CHN1 overexpression on the expression of p-Akt, p-GSK-3β, and Snail. Three independent experiments were performed, and GAPDH and β-tubulin were used as the internal controls

The high expression of CHN1 was observed in the metastatic lymph nodes of cervical carcinoma tissue (Fig. 1d). Since EMT is one of the key elements for cancer metastasis, the expression of CHN1 and one of the key EMT transcription factors, Snail, was detected in 24 serial-sliced CC samples to assess the correlation between these two proteins. It showed that, both were stained in brown with similar localizations in tumor samples (Fig. 5a), which implied that the level of Snail might related to the expression of CHN1 during cervical cancer development. The expression of another two crucial markers of EMT, epithelial marker E-cadherin and mesenchymal marker vimentin, were further analyzed in CHN1 downregulated SiHa cells to confirm the connection between EMT and CHN1. IF staining showed that, compared to the control, increased E-cadherin and decreased vimentin were observed in CHN1-interfered cells (Additional file 2: Fig. S1d).

qRT-PCR analysis showed that increased *CHN1* gene expression in SiHa and HeLa cells decreased that of genes corresponding to E-cadherin and β-catenin, but increased the expression of those corresponding to vimentin, fibronectin, and Snail (Fig. 5b). Similarly, western blotting showed that overexpression of CHN1 not only increased the expression of Snail, fibronectin, and vimentin, but also decreased that of E-cadherin and β-catenin, at the protein level (Fig. 5c). However, when CHN1 was disrupted, the expression of these EMT-related markers showed the opposite pattern (Fig. 5d). Activated Akt has been reported to be essential for the induction of EMT through the inhibition of GSK-3β, which leads to the stabilization and nuclear localization of Snail to trigger cell migration and EMT [30]. In this study, several key factors involved in the Akt/GSK-3β/Snail pathway were detected by western blotting, and the results showed that the expression of phosphorylated Akt, phosphorylated GSK-3β, and Snail was enhanced when CHN1 was upregulated (Fig. 5e). To further confirm that the effect of CHN1 was mediated by the activation of the Akt/GSK-3β/Snail pathway, the expression of these three proteins was tested again in the presence of the PI3K inhibitor LY294002. The levels of phosphorylated Akt, phosphorylated GSK-3β, and Snail were effectively reduced by LY294002 treatment (Fig. 5e).

## Discussion

The progression of cervical cancer is a multifactor and multistep process. Even though some progress has been made in cervical screening and diagnostic techniques, as well as in CC vaccines, it is still one of the major gynecologic malignancies worldwide, and an effective way to restrain CC development and inhibit relapse and metastasis needs to be determined.

In this study, the carcinogenesis of CHN1 was investigated to determine its role in the occurrence and development of cervical carcinomas. We found that overexpression of CHN1 in CC was related to a higher degree of metastasis, pathological stage, and reduced overall survival rate. Further in vitro functional studies demonstrated that the metastasis and tumorigenesis of SiHa and HeLa cells could be significantly enhanced by the increased level of CHN1. In contrast, an inhibitory effect was observed when CHN1 expression was interrupted, consistent with Liu’s previous finding that knockdown of CHN1 reduced aggressive cervical cancer cell behaviors [29]. Experiments on nude mice in vivo also supported the finding that higher levels of CHN1 promoted tumorigenesis, while lower levels inhibited tumor size. One of the main reasons for recurrence and death in CC patients is distant metastasis, for example in pelvic lymph nodes [31–33]. EMT plays a crucial role during lymph node metastasis in cervical cancer [34]. It has been reported that increased tumor progression, invasion, metastasis, and distortion of epithelial integrity can be observed if the primary cervical cancer is coupled with the occurrence of EMT [35]. Therefore, it is important to test whether EMT is involved in the tumorigenesis of CHN1. According to our data, in SiHa and HeLa cells, increased expression of CHN1 promoted the downregulation of epithelial markers, but induced the upregulation of mesenchymal markers, at both the RNA and protein levels. However, the expression of these EMT markers was significantly reversed when CHN1 levels were disrupted in CC cells.

In addition, the location of CHN1 was found to be related to the position of Snail, a key EMT principal transcription factor, by IHC staining of lymph nodes with CC metastases. The expression of Snail was regulated by CHN1 levels; increased expression of CHN1 correlated with high Snail levels, while decreased expression of CHN1 was associated with low Snail levels.

Activation of the Akt/GSK-3β/Snail pathway is involved in the occurrence of EMT in cervical cancer [36, 37]. Our results further showed that the levels of phosphorylated Akt, phosphorylated GSK-3β, and Snail were increased when the protein level of CHN1 was strongly enhanced, and the same tendency occurred when CHN1 was attenuated, suggesting that CHN1 might be required for the activation of Akt/GSK-3β/Snail to promote metastasis in cervical carcinoma.

## Conclusions

CHN1 overexpression promoted the proliferation, migration, and invasion of CC cells in vitro. In addition, CHN1 overexpression was significantly associated with a high degree of metastasis, low survival rate, and poor prognosis in CC patients. CHN1 could induce EMT through the Akt/GSK-3β/Snail pathway, as well as promote metastasis and CC progression. Thus, CHN1 can be considered as a novel marker of cervical carcinoma for the clinical diagnosis of metastasis and poor prognosis in CC patients. CHN1 is a promising target for further investigation to improve CC diagnosis and treatment.

## Supplementary Information

**Additional file 1:** **Table S1.** The specific primers used in our experiments.

**Additional file 2:** **Fig. S1.** Cellular localization of CHN1 (red) in SiHa cells and CC tissues. a IF staining of CHN1 in SiHa cells. Red signal represents CHN1, and blue signal represents nuclei (DAPI). CHN1 was mainly localized in the cytoplasm (magnification, 600×). b Representative CHN1 staining in CC tissue. CHN1 was expressed in the cytoplasm of CC tissue samples (original magnification, 200×). c Evaluation criteria of IHC staining to define the expression level of CHN1 in CC tissues. No expression of CHN1 was defined as negative (−), less than 50% expression of CHN1 was defined as positive (+), and more than 60% expression of CHN1 was defined as strongly positive (++) (original magnification, 40×). d Increased expression of E-cadherin and decreased expression of Vimentin were detected after down-regulating CHN1 expression in SiHa cells. DAPI (blue) was added to stain the nuclei (original magnification, 600×).

## Data Availability

The data generated during this study are included in this published article.

## Abbreviations

CHN1: α-Chimaerin gene
EMT: Epithelial-mesenchymal transition
CC: Cervical carcinoma
TMA: Tissue microarray
H&E: Hematoxylin and Eosin
IF: Immunofluorescence
IHC: Immunohistochemistry
qRT-PCR: Quantitative reverse transcription PCR
shRNA: Short hairpin RNA
