# Supplementary material for: CHN1 promotes epithelial–mesenchymal transition via the Akt/GSK-3β/Snail pathway in cervical carcinoma
Source: J Transl Med. 2021 Jul 8;19:295. doi: 10.1186/s12967-021-02963-7 (PMC8264971; doi:10.1186/s12967-021-02963-7)
Supplement: Supplementary file 1 — Additional file 1: Table S1. The specific primers used in our experiments. [file 12967_2021_2963_MOESM1_ESM.docx]

| **Gene** | **Primer sequences (5’-3’)** |
| --- | --- |
| CHN1 | F: GGAGCTACCTCATCCGGGAG  R: TGTGTCTCTTTCAGGACTGGCA |
| E-cadherin | F: TGCCCAGAAAATGAAAAAGG  R: GTGTATGTGGCAATGCGTTC |
| β-catenin | F: AAAATGGCAGTGCGTTTAG  R: TTTGAAGGCAGTCTGTCGTA |
| Vimentin | F: GAGAACTTTGCCGTTGAAGC  R: GCTTCCTGTAGGTGGCAATC |
| Fibronectin | F: ATGGAGGAAGCCGAGGTT  R: AGCGGTTTGCGATGGTAC |
| Snail | F: ATCGGAAGCCTAACTACAGC  R: CAGAGTCCCAGATGAGCATT |
| β-actin | F: CATGTACGTTGCTATCCAGGC  R: CTCCTTAATGTCACGCACGAT |

Table S1. The specific primers used in our experiments
