# Supplementary figures and images for: CHN1 promotes epithelial–mesenchymal transition via the Akt/GSK-3β/Snail pathway in cervical carcinoma
Source: J Transl Med. 2021 Jul 8;19:295. doi: 10.1186/s12967-021-02963-7 (PMC8264971; doi:10.1186/s12967-021-02963-7)

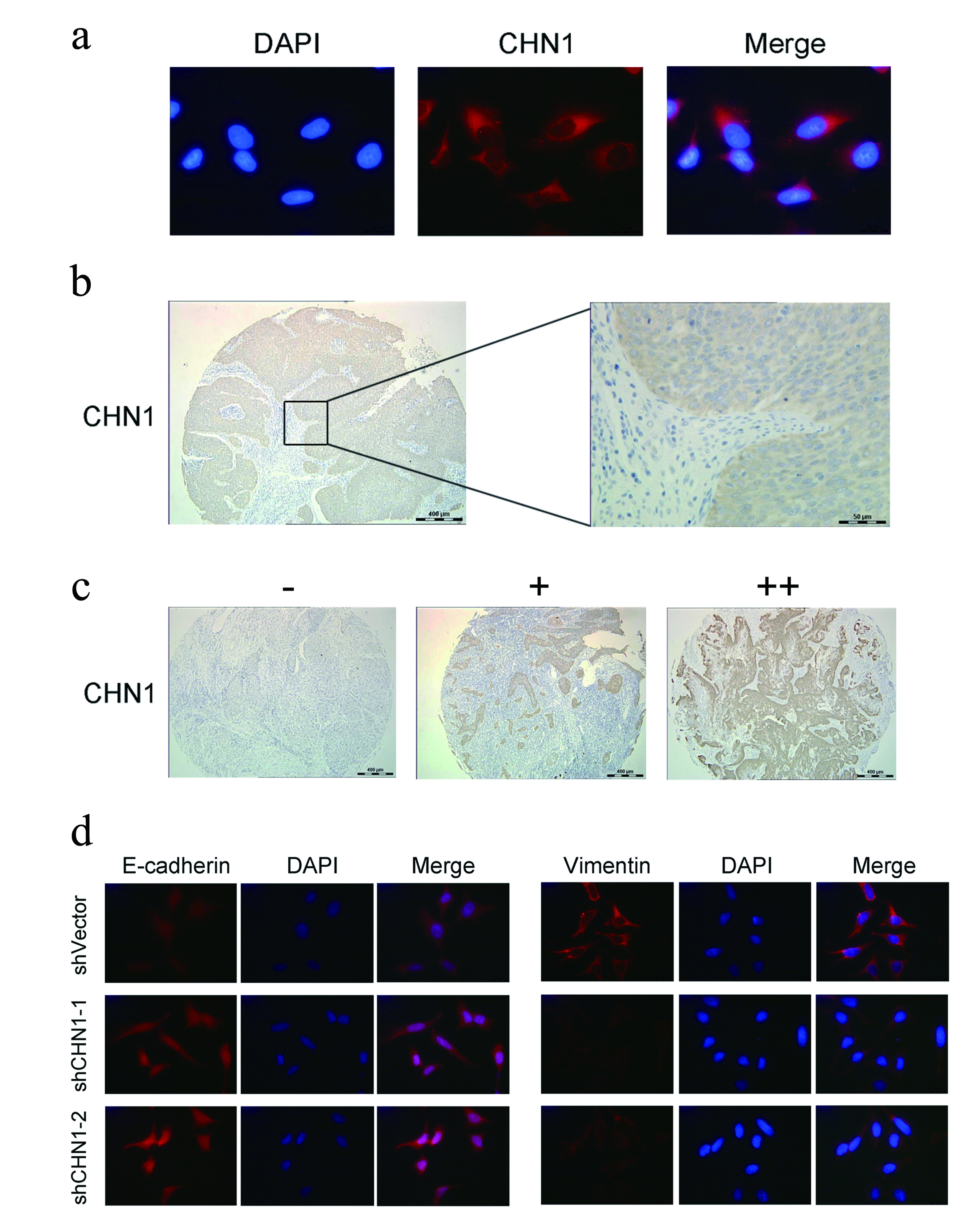

Supplement: Supplementary file 2 — Additional file 2: Fig. S1. Cellular localization of CHN1 (red) in SiHa cells and CC tissues. a IF staining of CHN1 in SiHa cells. Red signal represents CHN1, and blue signal represents nuclei (DAPI). CHN1 was mainly localized in the cytoplasm (magnification, 600×). b Representative CHN1 staining in CC tissue. CHN1 was expressed in the cytoplasm of CC tissue samples (original magnification, 200×). c Evaluation criteria of IHC staining to define the expression level of CHN1 in CC tissues. No expression of CHN1 was defined as negative (−), less than 50% expression of CHN1 was defined as positive (+), and more than 60% expression of CHN1 was defined as strongly positive (++) (original magnification, 40×). d Increased expression of E-cadherin and decreased expression of Vimentin were detected after down-regulating CHN1 expression in SiHa cells. DAPI (blue) was added to stain the nuclei (original magnification, 600×). [file 12967_2021_2963_MOESM2_ESM.tif]
